# Competition between health maintenance organizations and nonintegrated health insurance companies in health insurance markets

**DOI:** 10.1186/s13561-015-0073-3

**Published:** 2015-11-25

**Authors:** Edmond Baranes, David Bardey

**Affiliations:** 1 University of Montpellier (Lameta & Labex Entreprendre), 163 rue Auguste Broussonnet, Montpellier, 34090 France; 2 University of Los Andes, Cede, Bogota, Colombia; 3 Toulouse School of Economics (Gremaq), Toulouse, France

**Keywords:** L42, I11, Vertical integration, Health maintenance organizations, Competition policy

## Abstract

This article examines a model of competition between two types of health insurer: Health Maintenance Organizations (HMOs) and nonintegrated insurers. HMOs vertically integrate health care providers and pay them at a competitive price, while nonintegrated health insurers work as indemnity plans and pay the health care providers freely chosen by policyholders at a wholesale price. Such difference is referred to as an input price effect which, at first glance, favors HMOs. Moreover, we assume that policyholders place a positive value on the provider diversity supplied by their health insurance plan and that this value increases with the probability of disease. Due to the restricted choice of health care providers in HMOs a risk segmentation occurs: policyholders who choose nonintegrated health insurers are characterized by higher risk, which also tends to favor HMOs. Our equilibrium analysis reveals that the equilibrium allocation only depends on the number of HMOs in the case of exclusivity contracts between HMOs and providers. Surprisingly, our model shows that the interplay between risk segmentation and input price effects may generate ambiguous results. More precisely, we reveal that vertical integration in health insurance markets may decrease health insurers’ premiums.

## Background

Even though few articles analyze the consequences of vertical integration in the health insurance sector in theoretical terms,^1^ vertical integration is often presented as an efficient remedy for reducing or containing increasing health care expenditures. First, vertical integration allows insurers to reduce providers’ moral hazard and transaction costs. Second, as pointed out in Cutler, McClellan and Newhouse [1] and Melnick et al. [2] Health Maintenance Organizations (HMOs) negotiate lower prices with health care providers. Third, in the US context, HMO penetration has been associated with increased competition in the health insurance sector [3]. However, in spite of these optimistic views it is known that vertical integration can lead to anti-competitive situations [4]. More precisely, vertical integration may create incentives for foreclusion, increase providers’ (upstream) market concentration and consequently raise the input prices for nonintegrated insurers (downstream firms): the so-called “raise rivals’ cost” effect [5]. In the health insurance sector vertical integration may also generate risk segmentation among policyholders. Indeed, policyholders value the diversity of the health care providers that their health insurance plan provides and this value naturally increases with the probability of disease. Roughly speaking, policyholders characterized by a high probability of disease tend to prefer to be affiliated with nonintegrated health insurance companies that do not restrict their choice of health care provider.^2^

From an empirical perspective, it is difficult to disentangle these effects and, in particular, to understand if HMOs’ lower costs are due to risk segmentation, a raise rivals’ cost effect, or to the previously mentioned positive arguments. At this stage of the analysis it is worth noting that when taken separately the raise rivals’ cost effect and the risk segmentation effect work in the same direction and should yield an increase in premiums charged by nonintegrated health insurance companies. The goal of this article is to understand the interplay between these two effects, which can yield ambiguous results, as will be shown later on.

In this article, we provide a simple theoretical model in order to analyze how the number of HMOs affects the premiums charged by nonintegrated health insurers. We use the traditional framework of vertical integration provided by Salinger [6] to model competition where many HMOs and many nonintegrated health insurance companies participate. More precisely, policyholders choose between nonintegrated health insurance companies and HMOs and we assume perfect competition within the nonintegrated health insurers market, as well as within the HMOs market. Moreover, we consider that both markets are submitted to a community rating regulation. Therefore, insurers have to charge premiums according to the average risk of their policyholders, yielding a regulatory adverse selection [7].

Due to the perfect competition assumption, at equilibrium both types of insurer contract, i.e. nonintegrated health insurers and HMOs contracts, are charged at their respective marginal costs. Nevertheless, the marginal costs of nonintegrated health insurers and HMOs differ in two respects: the price paid to health care providers, which may involve a raise rivals’ cost effect, and the average health risk of their respective policyholders, i.e. the adverse selection due to the risk segmentation mentioned above. Concerning the first difference and following Salinger [6], we suppose that vertical integration allows HMOs to pay for the health care delivered by affiliated providers at their marginal cost, whereas nonintegrated health insurance companies have to pay a wholesale price to health care providers, which is by nature higher than the marginal cost. To capture the second difference we add an endogenous risk segmentation to Salinger’s framework between nonintegrated health insurance companies and HMOs. *Ceteris paribus*, policyholders with a higher risk of disease prefer to be insured with nonintegrated health insurance companies that do not restrict access to health care providers. Roughly speaking, this risk segmentation generates an adverse selection phenomenon since nonintegrated insurers’ policyholders are characterized by a higher probability of falling ill.

Following Salinger [6], we consider an exogenous market structure and provide some comparative static analyses in order to focus on the effects of the number of HMOs on health insurance premiums (nonintegrated health insurance companies and HMOs’ premiums).^3^ Our model attempts to understand the interplay between risk segmentation and health care price effects. First, we show that the number of HMOs affects the equilibrium allocation only when HMOs and providers form exclusive contracts since vertical integration increases concentration in the providers’ market, i.e. fewer providers are available for policyholders who choose nonintegrated insurers. Second, without assuming that the presence of HMOs increases competitive pressure in the health insurance market, our results show that the penetration of HMOs can, in some cases, reduce nonintegrated insurers as well as HMOs’ premiums. The model allows us to determine that the level of health care marginal cost constitutes a key factor in vertical integration welfare consequences for health insurance markets. This result departs from Gaynor and Ma (Gaynor, M and Ma, A: Insurance, vertical restraints, and competition, unpublished), who show that the reduced choice implied by exclusive contracts is detrimental to consumer surplus.

The game between health insurers and health care providers has already been thoroughly analyzed in (Gaynor, M and Ma, A: Insurance, vertical restraints, and competition, unpublished), Ma [8], Gal-Or [9, 10] and, more recently in Douven et al. [11] and Bardey and Bourgeon [12]. These articles endogenously determine the nature of the contracts that emerge at an equilibrium between hospitals and health insurers, but they rule out the risk segmentation effect. In contrast, we introduce a risk segmentation effect and take the type of contract as well as the market structure to be exogenous. Bardey and Rochet [13] take into account some risk segmentation but they apply it to the competition between HMOs and PPOs, assuming that PPOs provide a bigger network. Differently, our analysis does not really distinguish between HMOs and PPOs but focuses on risk segmentation and its consequences for, and interplay with, the raise rivals’ cost effect. We claim that our result may contribute to an explanation of the contrasting results provided in the empirical literature. In particular, we do not need the traditional positive positive arguments usually attributed to HMOs - such as that of the implementation of non-price rationing strategies to reduce providers’ moral hazard or an increase of competition in health insurance market - to exhibit that HMOs competition may contribute to lower nonintegrated insurers’ premiums and therefore tends to reduce HMOs’ marginal cost.

The rest of the paper is organized as follows. “The model” section presents the model. “Equilibrium analysis” section gives the outcome of the equilibrium and its properties are discussed. We conclude in “Discussion and conclusions” section.

## The model

This section presents the theoretical model that contains two sets of health insurance companies: HMOs and non-integrated.

### The health insurance companies

In our model there are many nonintegrated health insurance companies, as well as many HMOs. We note *n* the number of HMOs among insurance companies (as we shall see later on, the number of nonintegrated health insurance companies does not matter). The difference between nonintegrated health insurance companies and HMOs is that the latter offer a restricted list of providers to policyholders, while the former allow policyholders to freely choose among the providers available in the health care market. For the purposes of simplicity, we assume that policyholders who choose HMOs can only obtain care from one provider (who belongs to this HMO). Moreover, we assume that nonintegrated insurers and HMOs do not bear any costs other than the price of the health care they pay to providers. We note *R* and *r* the price paid to providers by nonintegrated insurers and HMOs, respectively. Nonintegrated health insurance companies charge a premium *P*_*CI*_ to policyholders, and HMOs a premium *P*_*HMO*_. We assume perfect competition between nonintegrated health insurance companies, i.e. they sell their health insurance contract at a marginal cost. The same assumption is applied to HMOs.^4^ Moreover, it is worth noticing that nonintegrated health insurance companies and HMOs’ marginal costs both depend on the average risk of the policyholders who choose the insurance plans on the one hand, and on the price paid to providers on the other.

### The providers

Throughout the paper, we consider that the market structure defined by the number of HMOs and providers is exogenous. This assumption allows us to perform comparative static exercises with respect to the number of HMOs in order to shed light on the effect of vertical integration in health care markets.^5^ We denote by *J* providers who offer health care to patients. We suppose that each provider bears a constant marginal cost, noted as *c*. We consider a situation where HMOs sign exclusive contracts with providers; that is, a provider who works for an HMO cannot supply health care to patients who are not affiliated with that HMO. Consequently, for a given number of providers the prevalence of exclusive contracts reduces the diversity of providers available to policyholders who choose nonintegrated health insurance companies. Hence, since we have *n* HMOs and each HMO offers access to one provider, this implies that policyholders who choose nonintegrated health insurance companies have access to *K*=*J*−*n* providers when they fall ill. Moreover, for simplicity, we consider a situation of single-homing in which providers affiliated with an HMO cannot offer health care to patients insured through another insurance company. By contrast, when vertical integration does not involve exclusive contracts, i.e. non-exclusive contracts are formed between HMOs and providers, then the number of providers available to patients affiliated with nonintegrated health insurance companies remains *K*=*J*. In other words, in such a case even providers who belong to HMOs are available to nonintegrated insurers’ policyholders.

Following Salinger [6], exclusive contracts between HMOs and affiliated providers lead to a crucial difference between both types of insurance plan. Nonintegrated health insurance companies pay providers for health care at a wholesale price *R* that includes a markup based on *c*, whereas HMOs, thanks to their vertical structure, obtain health care at marginal cost *c*, that is *r*=*c*.^6^ Finally, we consider competition between the independent providers using a circular location model. We normalize the length of the circle to 1 and we note provider *i*’s location by *x*_*i*_∈{1,…,*J*−*n*}, where *J*−*n* providers are located at an equal distance from one another.

### The policyholders

We assume that policyholders can only obtain health care when they become ill by purchasing a health insurance contract. We assume that policyholders benefit from full insurance coverage and do not pay any out-of-pocket expenses. Following Gal-Or [9, 10], we assume that policyholders are differentiated according to two dimensions. The first of these is an *ex ante* distribution of their probability of disease, noted by *θ*, which is uniformly distributed over [0,1] and corresponds with the probability of health care consumption. The second dimension is an *ex post* uniform distribution over a circle of length 1 that reflects the type of disease that affects policyholders when they become ill. The *ex post* address of policyholders is noted by *x*, and we assume a linear transportation cost, noted *t*. This *ex post* differentiation captures the following idea: when policyholders choose their health insurer (nonintegrated health insurance company or HMO) they do not know *ex ante* which kind of disease they will suffer from if they become ill and, consequently, do not know which is the best provider for treating their disease. This *ex post* differentiation can be interpreted as a preference for diversity under the veil of ignorance and yields a vertical differentiation according to the type of health insurer chosen. Roughly speaking, policyholders characterized by a high probability of being sick, *ceteris paribus*, prefer nonintegrated health insurance companies rather than HMOs due to the diversity of providers to which they have access via the former. Patients may bear a higher expected transportation cost by being insured with an HMO because the provider to which they have access is not necessarily the most suitable according to the disease captured by their address *x*. For the sake of simplicity, we assume that *ex ante* and *ex post* distributions are independent. The probability *θ*, which is supposed to be observable by insurers, is the key parameter that determines the risk segmentation among policyholders among both HMOs and nonintegrated health insurance companies.

The policyholders’ expected utility function can be written:

(1) $$ U=u+2\theta y\int_{0}^{1/2y}(v-tx)dx-P.   $$

The first term of the utility function represents the gross utility that an individual can obtain without an insurance plan. It can be viewed as the expected utility that an individual can obtain in the absence of insurance. The second term measures the benefit that a policyholder characterized by a probability *θ* derives from the insurance plan. Thanks to their plan, when they become ill they benefit from health care that gives a utility *v*, minus the expected transportation cost according to the provider they are allowed to choose. This expected transportation cost mainly depends on the number *y* of providers accessible and the probability of disease *θ*. Finally, policyholders have to pay a premium *P* to be insured.^7^

Remember that policyholders have access to only one provider if they choose an HMO (*y*=1) and to *K*=*J*−*n* when they are insured by a nonintegrated health insurance company. Consequently, the expected utilities for policyholders affiliated with a nonintegrated health insurance company and an HMO are respectively:

(2) $$ U_{CI}\left(\theta \right) =u+2\theta K\int_{0}^{1/2\left(J-n\right) }(v-tx)dx-P_{CI}   $$

and,

(3) $$ U_{HMO}\left(\theta \right) =u+2\theta \int_{0}^{1/2}(v-tx)dx-P_{HMO},   $$

where *P*_*CI*_ and *P*_*HMO*_ denote the premium charged by nonintegrated health insurance companies and HMOs, respectively.

Hence given a probability of disease *θ*, a policyholder prefers to buy a health insurance contract from a nonintegrated health insurance company rather than from an HMO if

(4) $$ U_{CI}\left(\theta \right) \geq U_{HMO}\left(\theta \right).   $$

Let us denote as $\tilde {\theta }$ the marginal policyholder indifferent to the two types of health insurance plan. So the last inequality applies if and only if

(5) $$ \theta \geq \tilde{\theta}=\frac{\left(P_{CI}-P_{HMO}\right) 4K}{t\left(K-1\right) }.   $$

A policyholder with a higher probability of disease than $\tilde {\theta }$ prefers to choose a nonintegrated health insurance company in order to enable choice of provider if they become ill, even if they have to pay a higher premium. We can observe that $\tilde {\theta }$ decreases with *K* and consequently increases in *n*. In other words, at this stage of the game nonintegrated health insurance companies obtain a higher market share when they provide a greater diversity of providers, i.e. $1-\tilde { \theta }$ increases. Note that their market share also increases with *t*, which is an inverse measure of the substitutability between the two types of insurance plan.

## Equilibrium analysis

We apply the standard sequential game that is used in the vertical integration literature to characterize the equilibrium. In the first stage we determine the wholesale price at the upstream level (the provider market). In the second stage we derive the equilibrium prices at the downstream level (i.e. the insurance sector). The game is solved backwards.

### Downstream competition

At the downstream level insurers compete à la Bertrand. As already mentioned, we assume a perfect competition outcome in both markets, the market of nonintegrated health insurance companies and the market of HMOs. In other words, we assume perfect competition and that no-loading factors matter in both health insurance markets. Moreover, we consider a community rating regime in which premiums reflect the average risk of policyholders affiliated with the same type of insurer.^8^ Therefore, in the nonintegrated insurers market premiums *P*_*CI*_ are equal to their marginal cost:

(6) $$ P_{CI}=\left(\frac{1+\tilde{\theta}}{2}\right) R,   $$

where $(1+\tilde {\theta })/2$ represents the average probability of disease for policyholders who choose nonintegrated insurers, while *R* is the price of the health care paid by nonintegrated insurers to providers.

*Ex ante*, policyholders do not know their *ex post* address (the type of disease they will suffer from if they become ill), hence when they choose their health insurance contract they have no particular preference for a specific provider. Behind this veil of ignorance there is no differentiation in the HMO market even though the “good” sold is different from an *ex post* perspective. Consequently, HMOs’ premiums are equal to their marginal costs: the health care marginal cost *c*, weighted by the average probability of HMO policyholders ($\tilde {\theta }/2$):

(7) $$ P_{HMO}=\frac{\tilde{\theta}}{2}c.   $$

The equilibrium in the subgame at the downstream level can be directly derived from the marginal policyholder definition and premiums. For a given price *R*, the health insurance sector equilibrium is given by:

(8) $$\begin{array}{@{}rcl@{}} P_{CI} &=&\frac{1}{2}R\left(1+\frac{2KR}{2cK-2KR+(K-1)t}\right),  \\ P_{HMO} &=&\frac{cKR}{2cK-2KR+(K-1)t},\notag \\ \tilde{\theta} &=&\frac{2KR}{2cK-2KR+(K-1)t}. \notag \end{array} $$

#### **Remark1**.

At this stage both premiums increase in the wholesale price *R*.

*Ceteris paribus*, at the given risk segmentation an increase in the wholesale price increases the premium of nonintegrated insurers. Consequently, HMOs become more attractive, in turn reinforcing the risk segmentation effect ($\tilde {\theta }$ increases in *R*). Both premiums increase in *R*.

### Upstream competition

Only a fraction ($1-\tilde {\theta }$) of policyholders choose their providers freely. Thus, according to the *ex post* uniform distribution address, each provider is actually in a monopolistic competition context where they must deal with a demand equal to $\left (1-\tilde {\theta } ^{2}\right) /2K$. Therefore, each provider *j* seeks to maximize:

$$\underset{R_{j}}{Max\Pi =}\left(R_{j}-c\right) \left(\frac{1-\tilde{\theta} ^{2}}{2K}\right), $$

where *R* is the average price paid to providers and $\tilde {\theta }$ is given by (8). We have $R=\overset {J}{\sum }R_{j}/K$. According to the uniform distribution of providers, they all obtain identical market shares. Consequently, the average is a nonweighted sum.The first-order condition gives:

(9) $$ \frac{\tilde{\theta}}{K}\frac{\partial \tilde{\theta}}{\partial R}(R_{j}-c)= \frac{1-\tilde{\theta}^{2}}{2},   $$

which, assuming a symmetric equilibrium, can also be written:

(10) $$ \frac{R^{\ast }-c}{R^{\ast }}=\frac{1-\tilde{\theta}^{2}}{2\tilde{\theta}^{2} }\frac{K}{\mu },  $$

where *μ*, which is given by $\left (\partial \tilde {\theta }(R^{\ast })/\partial R\right) \ast \left (R^{\ast }/\tilde {\theta }\right) $, represents the elasticity of the marginal policyholder with respect to the average price paid to physicians and *R* ^∗^ denotes the price charged by providers at the symmetric equilibrium of this subgame.

#### **Proposition1**.

In equilibrium, the provider markup directly: ^9^

- i) increases with the number of providers available for nonintegrated health insurance companies’ policyholders;
- ii) decreases with the elasticity of the wholesale price.

Equation (9) looks like the traditional formula obtained in Cournot’s oligopoly framework in the sense that the provider markup is inversely related to price elasticity. Nevertheless, significant differences may be noted. The first point of Proposition 1 (with respect to the number of providers) may be interpreted as an induced demand effect from providers when their control variable is price and not quantity [14]. When the number of providers increases, the diversity supplied by nonintegrated health insurance companies also increases. *Ceteris paribus*, their demand function shifts upwards, so the number of policyholders who can freely choose their provider increases, thus allowing providers to increase their markup. Actually, a positive externality occurs between providers due to the effect on the demand for nonintegrated health insurance companies. When the marginal policyholder is extremely price sensitive this demand effect obviously reduces the providers’ ability to increase their markup.

#### **Proposition2**.

If an interior equilibrium occurs, i.e. $\tilde {\theta }<1$, the profit of the providers is decreasing with *K* (is increasing in *n*).

#### *Proof*.

See Appendix. □

According to our specifications, Proposition 2 reveals that the competition effect dominates the markup effect. We now turn to an analysis of the properties of our equilibrium.

### Properties of the equilibrium

Instead of directly analyzing variations of *R* and $\tilde {\theta }$ with respect to *K*, the market share variation of HMOs with respect to *K* can be understood thanks to the following decomposition:

(11) $$ \frac{d\tilde{\theta}}{dK}=\frac{\partial \tilde{\theta}}{\partial K}+\frac{ \partial \tilde{\theta}}{\partial R}\frac{\partial R}{\partial K}.  $$

The first term of the RHS is called the diversity effect and is a direct consequence of risk segmentation. As described previously, if nonintegrated health insurance companies supply greater diversity (in terms of providers available) then the HMO market share decreases. The second term is the wholesale price effect. It captures the impact of the health care price paid by nonintegrated health insurance companies to physicians on the existing risk segmentation. Hence the higher the price paid by nonintegrated health insurance companies to providers, the higher the premium charged by nonintegrated health insurance companies to their policyholders and the lower their market share.

#### **Remark2**.

If the HMO market share increases with *K*, then *R* also increases with respect to *K* (decreases with respect to *n*).

As $\tilde {\theta }$ decreases with respect to *R* while the direct effect means that $\partial \tilde {\theta }/\partial K<0$, we have $d\tilde {\theta }/dK>0$, if and only if *∂* *R*/*∂* *K*>0. In the Appendix we define a threshold value for *c*, allowing us to give the following Proposition 3.

#### **Proposition3**.

The HMO market share increases with *K* (decreases with *n*) if $c<\widetilde {c}$.

#### *Proof*.

See Appendix. □

There are two countervailing effects at work: the input price and diversity (or risk segmentation) effects mentioned. In equilibrium, for small values of *c*, we show that the HMO market share increases with the number of physicians available in the market. In other words, when the health care marginal cost is low enough, instead of losing market share because the diversity supplied by nonintegrated health insurance companies increases and makes them more attractive, HMO market share increases because providers charge a higher price to nonintegrated health insurance companies who are obliged to increase premiums for their policyholders. As HMOs benefit from health care at its marginal cost, the difference *P*_*CI*_−*P*_*HMO*_ increases such that it dominates the increase of the diversity supplied by conventional insurers.

Based on Remark 2 and Proposition 3, we can summarize the results obtained in the following corollary:

#### **Corollary1**.

For small values of *c* (i.e. $c<\widetilde {c}$), *R*, *P*_*CI*_ and *P*_*HMO*_ are increasing in *K* and decreasing in *n*.

Variations of premium *P*_*CI*_ become intuitive: In equilibrium, for small values of *c*, $\tilde {\theta }$ and *R* increase in *K*. As premiums charged by nonintegrated insurers increase with *R* and $\tilde {\theta }$, *P*_*CI*_ also increases in *K* and decreases in the number of HMOs (*n*). This corollary reveals that when the level of the health care marginal cost is low enough, an increase in the number of HMOs (which corresponds to a decrease in the diversity supplied by nonintegrated health insurance companies), is ambiguous for policyholders welfare. On the one hand, all policyholders pay lower health insurance premiums when the number of HMOs increases. On the oher hand, policyholders benefit from a reduced diversity of providers. Proposition 3 and corollary 1 thus reveal a trade-off in the case of exclusive contracts between diversity of providers and the fact that the markup of providers may decrease with the number of HMOs (for $c<\widetilde {c}$). Actually, competition between HMOs and nonintegrated health insurance companies generates two inefficiencies: a wholesale price above the marginal cost and transportation costs generated by *ex post* differentiation.

In the literature that deals with vertical integration the main issue under analysis is the occurrence of some “raise rivals’ cost” effects. Since more HMOs reduce the number of health care providers available, the wholesale price paid by nonintegrated insurers to providers increases. This increase in wholesale price increases the nonintegrated insurers’ marginal cost and thus their premiums. In our framework the nature of the relationship between vertical integration and “the rivals’ cost” is different. Vertical integration reduces the number of providers, therefore decreasing the diversity generated by nonintegrated health insurance companies. Nevertheless, for $c<\widetilde {c}$, the reduction in diversity also decreases the wholesale price, which *ceteris paribus* reduces the nonintegrated insurers’ marginal costs. Therefore, for a low health care marginal cost our results are exactly the opposite to the so-called “raise rivals’ cost” effect. The greater the number of HMOs the more the nonintegrated health insurance companies’ marginal cost, and consequently their premiums, decrease.

Concerning the consequences of a contract’s nature, i.e. exclusive *versus* non-exclusive contracts, our model allows us to make the following remark.

#### **Remark3**.

The vertical integration in health care markets does not affect the equilibrium allocation in the case of non-exclusive contracts between health insurers and health care providers.

In the case of non-exclusive contracts the number of providers available in the market remains *K*=*J*. Consequently, the number of HMOs (*n*) does not intervene in the equilibrium allocation as long as it does not imply increased competition intensity in the health insurance market, as is the case in our set-up.

Finally, it is worth highlighting that most of the time the negative correlation between premiums charged by nonintegrated health insurance companies and HMO penetration is explained by increasing competition in the health insurance sector [3]. In this article, without taking into account this possible effect, our results indicate that the premiums charged by nonintegrated insurers may decrease with the number of HMOs when they sign exclusive contracts with providers. Our results also sheds light on the ambiguous evidence obtained in the literature about the consequences of vertical integration for the health sector [15]. Roughly speaking, we point out that the health care marginal cost may be part of the different results obtained by empirical studies that deal with vertical integration and exclusive contracts in the health care sector.

## Discussion and conclusions

This model attempts to analyze the impact of vertical integration in the health insurance sector. As in Gaynor [15], for health care markets our results indicate that there is no definitive or unconditional conclusion concerning the consequences of vertical integration in health insurance markets. Nevertheless, our framework identifies some market structure parameters that can influence vertical integration consequences in the case of exclusive contracts between providers and HMOs. More precisely, our model reveals that the so-called raise rivals’ cost effect only occurs for a specific range of health care marginal cost. For smaller values, vertical integration can paradoxically lead to a reduction in nonintegrated insurers’ premiums.

This model could be extended in several ways. First, it would be interesting to focus on a situation in which some of the health insurers are public or not-for-profit [16]. Second, we assume that the market is covered on the policyholder side, which is not necessarily the case in practice. Therefore, our model could be modified to take into account an uncovered market and to introduce a universal service issue, as is the case in other sectors [17]. Finally, it is worth noting that even though our set up is devoted to an analysis of vertical integration in the health insurance sector, it could be applied to all other sectors in which vertical restraints imply a reduction in product diversity and where there exists sufficient heterogeneity among consumers with respect to diversity valuation. In such a case, our model should be slightly modified to take into account the fact that consumer willingness to pay does not necessarily follow a probability structure.

## Endnotes

^1^ See, for instance, the recent review of literature provided by Gaynor and Town [18].

^2^ Our model takes into account the risk segmentation that occurs between nonintegrated health insurance companies and HMOs. The adverse selection is then directly determined by the risk segmentation at work. In a two-sided market environment, Bardey and Rochet [12] consider a general risk distribution function in order to disentangle more deeply the risk segmentation and the adverse selection effects.

^3^ Bardey and Bourgeon [12] consider an endogenous market structure, but they do not tackle the risk segmentation issue.

^4^ This perfect competition assumption among HMOs makes sense since HMOs are identical from an *ex ante* perspective, even though from an *ex post* perspective HMOs are differentiated due to the providers’ differentiation. In other words, before falling ill policyholders do not have any preferences among providers.

^5^ See Bardey and Bourgeon [12] for an analysis in which vertical integration is endogeneous.

^6^ We do not model HMOs’ strategies such that non-price rationing mechanisms, which allow them to reduce providers’ moral hazard and therefore their costs. However, since we assume that HMOs’ marginal cost is lower than the wholesale price paid by nonintegrated insurers, we already take this advantage into account in a reduced form. Practically, the reduction of providers’ moral hazard within HMOs yields a lower marginal cost.

^7^ In the Salop model, the diversity value is measured by:

$$-2\theta {y_{0}^{1/2y}}txdx=-\frac{\theta t}{4y}. $$

Since the function −(*θ* *t*/4*y*) is increasing and concave in *y*, it is worth to note that this diversity value introduced in the Salop model can be interpreted as risk aversion toward transportation cost.

^8^ This assumption is natural according to the large market share of collective health insurance contracts in the United States. As pointed out in Handel et al. [19], the Affordable Care Act removes the ability of insurers to adjust their premiua according to individual health risk.

^9^ specify that it is a direct effect because *K* also appears in $\tilde { \theta }$ and in the price elasticity *μ*.

## Appendix

### Proof of Proposition 2

The comparative static of the provider profit function is given by

$$\frac{d\Pi }{dK}=\frac{\partial \Pi }{\partial R}\frac{dR}{dK}+\frac{ \partial \Pi }{\partial K}. $$

The envelope theorem implies that *∂* *Π*/*∂* *R*=0. It yields

$$\frac{d\Pi }{dK}=\frac{\partial }{\partial K}\left(\frac{1-\tilde{\theta} ^{2}(R,K)}{2K}\right) \equiv \Gamma (K), $$

with

$$\Gamma (K,t,c,R)=-1+\frac{4K^{2}R^{2}\left(t+K\left(2c-2R+t\right) \right) }{\left(2cK-2KR+(K-1)t\right)^{3}}. $$

Then the goal of the proof is to sign *Γ*(*K*) (the sign of *d* *Π*/*d* *K* being the same). It is worth to notice that for *t*=0, we have

$$\Gamma (K,t=0,c,R)=-8cK^{3}\left(c-2R\right) (c-R)<0. $$

Moreover, the function *Γ*(*K,t,c,R*) is decreasing in *t* if and only if

$$\begin{aligned} \frac{\partial \Gamma (K,t,c,R)}{\partial t}=4K^{2}&(1+K)R^{2}-3\left(K-1\right)\\ &\left(2cK-2KR+(K-1)t\right)^{2}\leq 0. \end{aligned} $$

In the case of an interior equilibrium we have $\tilde {\theta }<1$. Taking into account that

$$\tilde{\theta}=\frac{2KR}{2cK-2KR+(K-1)t}, $$

we have

$$\begin{array}{@{}rcl@{}} \tilde{\theta} &<&1\Leftrightarrow 2cK-2KR+(K-1)t>2KR \\ &\Leftrightarrow &\left(2cK-2KR+(K-1)t\right)^{2}>\left(2KR\right)^{2}. \end{array} $$

The previous inequality (with the condition *K*≥2) implies that

$$4K^{2}(1+K)R^{2}<3\left(K-1\right) \left(2cK-2KR+(K-1)t\right)^{2}, $$

so that

$$\frac{\partial \Gamma (K,t,c,R)}{\partial t}<0. $$

As *Γ*(*K,t*=0,*c,R*)<0 and the function *Γ*(.) is decreasing in *t*, we have *Γ*(*K,t,c,R*)<0 that prove *d* *Π*/*d* *K*<0. Q.E.D.

### Proof of Proposition 3

We find some conditions for *R* to insure that *θ* increases with respect to *K*. We are able to exhibit some values of the parameters (*c,t*) for which these conditions are checked in equilibrium (with *R*=*R* ^∗^).

Our proof is performed in two steps. First (Step 1) we determine the general condition that ensures that the HMOs’ market share increases with respect to *K*. Second (Step 2) we determine the conditions for parameter *c* such that *θ* increases with respect to *K*. According to (Step 1) and (Step 2) we can conclude that the HMO market share increases with respect to *K* if *c* is sufficiently low.

(Step 1) The marginal policyholder is defined by:

(12) $$ \widetilde{\theta }=\frac{2KR}{(2cK-2KR+(K-1)t)}.   $$

Note that the derivative of function $\widetilde {\theta }$ with respect to *R* is given by:

(13) $$ \frac{\partial \widetilde{\theta }}{\partial R}=2K\frac{2Kc+(K-1)t}{\left(2cK-2KR+(K-1)t\right)^{2}}.   $$

The maximization of each provider’s profit enables us to determine equilibrium price *R* ^∗^ which is paid by insurers. Then using (9), (12) and (13) we obtain *R* ^∗^ as a function of *K*, *R* ^∗^=*R*(*K*):

(14) $$ R(K)=\frac{6cK^{2}+3tK^{2}-3tK-4cK-H}{8K(K-1)},   $$

with

(15) $$ H=\sqrt{4c^{2}(K-2)^{2}K^{2}+4cK^{2}\left(K^{2}+K-2\right) t+K(K-1)^{2}(8+K)t^{2}}.   $$

Let us define the HMO market share as a function of *K* and *R*, $\widetilde {\theta }=\theta (\textit {K,R})$. Then substituting *R* with *R* ^∗^=*R*(*K*) into *θ*(*K,R* ^∗^) we are able to determine the equilibrium HMO market share as a function of *K*, $\widetilde {\theta } =\theta (\textit {K,R}(K))$. The differentiation of $\widetilde {\theta }$ with respect to *K* leads to the following expression:

$$\frac{d\widetilde{\theta }}{dK}=\frac{\partial \widetilde{\theta }}{ \partial K}+\frac{\partial \widetilde{\theta }}{\partial R}\frac{dR(K)}{dK}. $$

Then $\widetilde {\theta }$ increases with respect to *K* if and only if

$$\frac{\partial \widetilde{\theta }}{\partial K}+\frac{\partial \widetilde{ \theta }}{\partial R}\frac{dR(K)}{dK}>0, $$

which is equivalent to:

(16) $$ \frac{R^{^{\prime }}(K)K}{R(K)}>\frac{t}{2cK+tK-t}.   $$

Denote the function *f*(*R* ^∗^,*c,t,K*) such that:

(17) $$ f(R^{\ast },c,t,K)=\frac{R^{\prime }(K)K}{R(K)}-\frac{t}{2cK+tK-t}.   $$

Then the condition (16) basically becomes *f*(*R* ^∗^,*c,t,K*)>0.

The equilibrium price paid by insurers is given by (14). Substituting *H* into (14) and taking the derivative of (14) with respect to *K*, we obtain:

(18) $$ {\fontsize{6.8pt}{9.6pt}\selectfont{\begin{aligned} R^{\prime }(K)=\frac{ 3tK^{3}c+2K^{3}t^{2}-2c^{2}K^{3}-6t^{2}K^{2}+6t^{2}K-2t^{2}+4c^{2}K^{2}-3cK^{2}t-cKH }{4KH\left(K-1\right)^{2}}.  \end{aligned}}}  $$

From (14), by writing *H* as a function of *R*, we have:

(19) $$ H=K\left(-3t-4c+6cK+3tK-8RK+8R\right).   $$

Combining (19) with (18), (17) becomes:

$$f(R^{\ast },c,t,K)=\frac{N(R^{\ast })}{2H\left(K-1\right) R^{\ast }\left(cK+tK-t\right) }, $$

where

(20) $$ \begin{aligned} N(R^{\ast }) =&~16tK\left(K-1\right)^{2}R^{\ast 2}-2K\\ &\left(t\left(K-1\right)^{2}(3t+4c)-4c^{2}K^{2}\right) R^{\ast}  \\ &+\left(K-1\right) t(\left(K-1\right)^{2}t^{2}+2Kct(K-1)-4K^{2}c^{2})\\ &-8c^{3}K^{3}. \end{aligned}  $$

Since the denominator of *f*(*R* ^∗^,*c,t,K*) is always positive, we can focus on the sign of the numerator *N*(*R* ^∗^,*c,t,K*).

(Step 2) At this stage, we study the function *N*(*R*) to determine whether *f*(*R,c,t,K*) is positive or not. Due to the complexity of the expressions, we proceed in two steps: first, we study the function *N* with respect to *R*. Second, we determine the sign of *N* at *R* ^∗^.

Denote *Δ* the discriminant of function *N*(*R*) such that we have:

(21) $$ {\fontsize{9.1pt}{9.6pt}\selectfont{\begin{aligned} \Delta =&~64c^{4}K^{6}+384K^{4}t(K-1)^{2}c^{3}+32K^{2}t^{2}(7K^{2}-12K+2)\\ &(K-1)^{2}c^{2}-32K^{2}t^{3}(K-1)^{4}c-4Kt^{4}(7K-16)(K-1)^{4}  \end{aligned}}}  $$

Note that for *c*=0, the discriminant is negative (for *K*>2) and decreasing with respect to *c* (i.e. *Δ*<0 and *∂* *Δ*/*∂* *c*<0). Moreover, *Δ* is convex with *c*:

$$\begin{aligned} \frac{\partial^{2}\Delta (c)}{\partial c^{2}}=~&64K^{2}\left(7K^{2}-12K+2\right) \left(K-1\right)^{2}t^{2}\\ &+2304K^{4}\left(K-1\right)^{2}ct+768K^{6}c^{2}>0. \end{aligned} $$

We can conclude that there exists a value of *c*, noted $\widetilde {c}$, such that *Δ*=0 for $c=\widetilde {c}$. Then *Δ*<0 if $c<\widetilde {c}$ and in this case *N*(*R*) is positive, in particular when *R*=*R* ^∗^. We can then conclude that *∂* *θ*/*∂* *K*>0 if $ c<\widetilde {c}$.
